# Communicating radiation risk in healthcare: an ethical imperative amidst scientific uncertainty

**DOI:** 10.3389/fpubh.2026.1883274

**Published:** 2026-07-23

**Authors:** Faisal Alrehily

**Affiliations:** Department of Diagnostic Radiology, College of Applied Medical Sciences, Taibah University, Madinah, Saudi Arabia

**Keywords:** diagnostic radiography, healthcare ethics, informed consent, radiation dose, radiation risk communication

## Abstract

The integration of ionizing radiation into modern clinical medicine underscores a critical, yet frequently inadequately addressed, institutional responsibility: patient-centered risk communication. This review synthesizes current empirical literature on how radiation risks are articulated and understood across diagnostic and therapeutic settings, distinguishing between the low-dose diagnostic applications and high-dose therapeutic radiation. Evidence indicates that despite established ethical and regulatory mandates, effective risk dialogue remains severely compromised by entrenched public knowledge gaps, media-driven anxieties, patient stress, and a bidirectional miscalibration of risks, whereby routine imaging hazards are frequently overstated and therapeutic adverse effects underestimated. These patient-level vulnerabilities are further compounded by institutional constraints, including a historic lack of formalized communication training for radiographers and the persistent underutilization of validated visual tools. To resolve these friction points, this paper identifies evidence-based communicative strategies, favoring graded disclosure frameworks, plain-language environmental analogies, and visually optimized data representations utilizing natural frequencies over rigid, “one-size-fits-all” approaches.

## Introduction

Ionizing radiation is integral to modern medical practice, driving advancements in diagnostic imaging, cancer therapy, and minimally invasive interventions. While enhancing diagnostic accuracy and therapeutic precision, its use carries inherent risks (1). Diagnostic modalities—such as plain radiography, computed tomography (CT), and fluoroscopy—utilize comparatively low radiation doses to acquire anatomical or functional data. In contrast, therapeutic applications in oncology employ substantially higher doses to ablate malignant tissue. This divergence in dose and clinical objective yields distinct risk profiles, necessitating tailored paradigms for clinical decision-making and patient communication. Clear dialogue allows patients to weigh benefits against potential harms, enabling meaningful participation in their care. Developing robust frameworks for communicating radiation risk is critical not only for regulatory compliance but also for advancing patient-centered medical practice amid rapidly evolving technologies.

Effective communication of radiation-associated risks is an ethical imperative, serving as the foundation for informed consent, collaborative decision-making, and patient safety (2–6). This review critically synthesizes current evidence on patient-centered radiation risk communication. It draws a clear distinction between the communicative obligations inherent to diagnostic imaging, where exposure levels are modest and risk terminology must remain proportionate, and those required for therapeutic interventions, which involve substantially higher doses and necessitate strict analyses of risks and benefits. Ultimately, this synthesis yields recommendations grounded in evidence to guide clinical practice and optimize training curricula within radiological disciplines. To achieve this, searches were conducted across PubMed and Google Scholar using combinations of the following terms: “radiation risk communication,” “informed consent radiation,” “low dose radiation risk,” “patient radiation perception,” “radiographer communication training,” and “visual aids radiation.” Inclusion was guided by relevance to patient communication, clinical practice, and radiation risk, focusing on literature published in English.

### The uncertain risk of low radiation dose

A central challenge in communicating radiation risk arises from the scientific ambiguity surrounding low-dose exposures, particularly below 100 millisieverts (mSv)—the range commonly used in diagnostic procedures (7–12). To contextualize these doses, practitioners often compare them to natural background radiation, which averages approximately 2–3 mSv per year (0.2–0.3 rem/year) globally. Against this baseline, a standard chest X-ray delivers approximately 0.2 mSv (0.02 rem), while a single CT examination contributes roughly 5 mSv (0.5 rem). For most patients undergoing infrequent imaging, these values represent a modest incremental increase in their total radiation burden (13).

The biological effects of low-dose ionizing radiation remain debated (14–17). While high-dose exposures have well-documented health consequences, risks below 100 mSv lack definitive epidemiological evidence linking them to adverse outcomes. This uncertainty complicates efforts to provide precise risk estimates. Current assessments largely extrapolate from high-dose cohorts, such as atomic bomb survivors and nuclear accident victims, heavily relying on the linear no-threshold (LNT) model. The LNT model posits that any radiation dose carries some carcinogenic risk; however, its validity at low doses is contentious (17–20). While some large-scale studies report excess cancer risks at 100 mSv or less, suggesting the LNT model does not substantially overestimate harm (19), competing paradigms challenge this assumption. Specifically, the threshold model and the radiation hormesis hypothesis argue that very low doses may prompt protective biological responses, such as apoptosis-mediated elimination of genomically unstable cells (21). Given this scientific ambiguity, risk communication must be proportionate: acknowledging theoretical risks at diagnostic levels without using language that overstates harm, discourages clinically justified imaging, or induces undue anxiety.

Balancing these uncertainties requires careful ethical consideration. Proponents of the precautionary principle advocate for the disclosure of all risks, even hypothetical ones, emphasizing the ethical obligation to inform patients of any potential harm. Conversely, overstating these uncertainties may lead to unnecessary anxiety, the avoidance of beneficial medical procedures, and the erosion of trust in healthcare providers. A graded approach to risk communication, calibrated to the dose level, frequency of exposure, and clinical context, offers a practical and ethically sound framework. Under this model, patients receive more detailed risk information for procedures involving a higher dose, such as CT, than for examinations utilizing a lower dose, such as plain radiography.

Although cellular repair mechanisms often mitigate the biological risks associated with low diagnostic doses (22, 23), these risks still warrant careful consideration in patient communication. Ionizing radiation induces DNA damage, which is typically repaired efficiently under standard imaging exposures. However, cumulative or elevated doses—such as those from repeated imaging or complex interventional fluoroscopy—can overwhelm reparative pathways, increasing the probability of stochastic effects like carcinogenesis (24–27). For instance, a European survey found that while most patients undergoing recurrent CT examinations stayed within safe cumulative thresholds, approximately 0.5% accumulated doses exceeding 100 mSv. This subgroup, predominantly comprising oncology patients, underscores the need for robust dose management systems and heightened awareness among referring physicians (28). While the absolute lifetime cancer risk attributable to medical radiation is low for individuals, widespread utilization of imaging technologies amplifies this risk at the population level. Furthermore, deterministic effects—including cataracts, erythema, and epilation—may manifest after prolonged high-dose exposures during interventional procedures, such as embolization or cardiac catheterization (29–31).

Therapeutic uses of ionizing radiation represent a fundamentally different clinical context. Radiotherapy delivers doses magnitudes higher than diagnostic imaging to purposefully destroy malignant tissue. At these therapeutic levels, both stochastic and deterministic effects become clinically relevant, and the irradiation of surrounding healthy tissue is an unavoidable consequence. Treatment side effects range from acute reactions (e.g., fatigue, dermatitis, and mucositis) to late-stage complications (e.g., fibrosis, secondary malignancies, and organ dysfunction), depending on the targeted site (32–36).

Consequently, clinical decisions must center on a rigorous evaluation of the risk-benefit ratio, transparently weighing the potential for disease control against treatment-related dose. Informed consent for radiotherapy carries a distinctly heavier ethical weight than for diagnostic imaging. It mandates comprehensive disclosure of short- and long-term side effects, the probability of therapeutic success, and viable alternatives. Practitioners must ensure patients grasp both the rationale for treatment and its expected consequences, thereby facilitating genuinely autonomous decision-making (37).

### Barriers to risk understanding

Although the necessity of transparent risk communication is well established (3, 38–40), conveying the probabilistic, long-term, and context-dependent nature of radiation exposure remains clinically challenging. This difficulty stems primarily from the public's limited baseline understanding of ionizing radiation; many patients cannot distinguish it from non-ionizing sources or lack awareness of its biological mechanisms (41, 42).

Consequently, patients frequently miscalibrate the risks associated with clinical applications of ionizing radiation. While unfamiliar technical jargon typically causes individuals to overestimate the minimal hazards of routine imaging (43–45), evidence also points to the inverse: patients may underestimate the substantial risks of therapeutic interventions. This bidirectional misperception underscores that risk interpretation is highly context-dependent (43).

Media amplification of nuclear incidents further distorts these perceptions (41). Sensationalized coverage of events like the Fukushima disaster or isolated clinical overexposures cultivates disproportionate anxiety (46, 47), impeding practitioners' efforts to balance the low hazards of medical imaging against its immediate diagnostic value (48). As a result, patients struggle to weigh tangible clinical benefits against abstract, long-term oncologic risks (49).

Cognitive processing is also compromised by the acute stress of seeking medical care. The emotional burden of a potential diagnosis disrupts rational risk assessment and the retention of complex information (43, 50, 51). Furthermore, this cognitive vulnerability intersects with varying levels of health literacy and cultural paradigms, as societies demonstrate divergent baselines for risk tolerance and medical trust (52–55).

Institutional barriers further compound these patient-level factors. Practitioners frequently lack formalized training in risk communication (56–61). Radiographers often report uncertainty when formulating dose comparisons (e.g., equating CT scans to background radiation) fearing they might inadvertently alarm patients (41). However, the current evidence base is limited by its reliance on self-reported practitioner confidence rather than objective assessments of clinical communication quality. Compounding these deficits are severe time constraints that lead to rushed, incomplete explanations (41, 60), alongside the pervasive underutilization of validated visual aids that could otherwise bridge literacy gaps (57, 58, 60–62).

### Strategies for effective risk dialogue

Because risk dialogue directly influences informed decision-making and treatment adherence, standard “one-size-fits-all” protocols are increasingly recognized as inadequate (41, 63). Patient-centered frameworks that adapt to individual health literacy—utilizing plain language, visual tools, and relatable analogies—yield superior comprehension. Patients prefer a graded disclosure model, where high-dose procedures trigger more comprehensive discussions, ideally combining verbal explanations with supplementary written materials (64).

De-escalating technical jargon is foundational to this approach. Practitioners can anchor abstract metrics in familiar experiences by stating, for example: “The radiation from this CT scan roughly equals what you would naturally absorb from the environment over 3 years—it carries a negligible theoretical risk, but provides crucial diagnostic information.” Such analogies make esoteric dosimetric data tangible without trivializing patient anxieties (49, 54). In practical terms, practitioners may say, for example: “The radiation from this CT scan is roughly equivalent to what you receive from natural background sources over 3 years—it carries a very small theoretical risk, but the information it provides is essential for your diagnosis.” Such comparisons anchor abstract dose values in everyday experience without trivializing the patient's concern. When language barriers arise, certified medical interpreters remain essential to preserve this nuanced messaging (65–68).

Beyond simplification, effective dialogue requires personalization. Tailoring discussions to a patient's age, educational background, cultural context, and emotional state fosters deeper engagement (41, 42, 69). Active listening transforms these encounters from unilateral disclosures into bidirectional dialogues, consistently reducing patient skepticism more effectively than rigid, fact-based delivery (70). Integrating frequent comprehension checks and open-ended queries allows providers to rapidly identify and correct misconceptions (49, 71). Crucially, validating a patient's fear creates the psychological safety necessary for rational deliberation.

Visual representation also circumvents many numeracy barriers. Pictographs, bar charts, and interactive three-dimensional simulations reliably enhance the retention of probabilistic data, especially for populations with lower health literacy (72–74). To maximize their utility, visual aids must adhere to evidence-based design principles. Best practices include utilizing natural frequencies (e.g., “3 in 10,000” rather than “0.03%”), maintaining consistent denominators across risk comparisons, referencing familiar baselines like annual background radiation, pairing graphics with explicit textual summaries, and pre-testing materials with representative patient cohorts (72, 74, 75).

### Training and skill development

Addressing the communication gap requires structured pedagogical interventions within radiography education. A notable example is the FORECAST2 project, an experience-based co-design initiative aimed at enhancing empathic communication between therapeutic radiographers and breast cancer patients (76). The project revealed a critical dissonance: while patients sought personalized reassurance, radiographers felt underprepared to navigate the emotional weight of cancer-related fears. This insight catalyzed the development of the KEW (Know, Encourage, Warmth) Model, a framework designed to build patient confidence and systematically integrate empathy into clinical workflows. Although national evaluations confirmed the KEW Model's educational value for continuing professional development, its generalizability beyond breast radiotherapy cohorts necessitates further empirical validation across diverse clinical settings (76).

The findings revealed that while patients valued reassurance, clear explanations, and individualized care, radiographers often felt underprepared to handle emotionally charged conversations, particularly around the fear of cancer recurrence (76). Through a series of co-design workshops, participants developed the KEW Model for Empathic Communication, structured around three key principles: Know, Encourage, and Warmth. This model guides radiographers in building patient confidence, acknowledging emotional concerns, and providing supportive, personalized communication throughout the treatment process.

Delivery mechanisms for these competencies also dictate their success. Quasi-experimental data demonstrate that dynamic multimedia training modules yield superior knowledge retention and engagement regarding radiation safety compared to traditional, static text-based instruction (77). However, traditional knowledge-based testing is insufficient to capture true competency acquisition. Robust evaluation of these programs requires observational metrics of behavioral change in clinical practice, utilizing tools such as patient satisfaction scores and standardized patient simulations (78).

Recognizing these needs, professional societies increasingly mandate the integration of communication competencies into core radiographic practice (77, 79). The radiographer's role now extends beyond technical operator to include primary patient advocate—a dual mandate made even more critical by the integration of artificial intelligence and complex imaging algorithms. Consequently, communication training cannot be relegated to a single undergraduate module; it must be a continuous, iteratively assessed component of lifelong professional development to keep pace with evolving procedural and technological landscapes.

## Conclusion

The continuous advancement of radiological and therapeutic technologies necessitates a parallel evolution in how clinical risks are articulated to patients. Effective risk communication is not merely a bureaucratic prerequisite for regulatory compliance; it is a core pillar of ethical, patient-centered medicine that directly dictates the validity of informed consent, therapeutic compliance, and the mitigation of clinical anxiety. As this review establishes, navigating the scientific ambiguities inherent to low-dose radiation or the profound physiological impact of oncology treatments requires a definitive departure from paternalistic, standardized information dumping toward highly personalized, graded dialogue.

Dismantling the barriers to patient understanding demands changes at both the practitioner and the institutional level. Individually, practitioners must actively counteract public misperceptions and acute cognitive stress by anchoring abstract dosimetric data in clear environmental analogies and validated visual tools. Structurally, however, tactical communication is only as sustainable as the educational and operational frameworks that support it.

Ultimately, elevating the standard of care requires a structural re-engineering of radiography education, wherein interpersonal, emotional, and communication competencies are evaluated with the same pedagogical rigor as technical proficiency. By embedding co-designed, empathy-driven frameworks and interactive multimedia platforms into undergraduate curricula and lifelong professional development programs, the radiological disciplines can better support practitioners in their expanding mandates.
